# Supplementary material for: Pancreatic cancer prognosis is predicted by an ATAC-array technology for assessing chromatin accessibility
Source: Nat Commun. 2021 May 24;12:3044. doi: 10.1038/s41467-021-23237-2 (PMC8144607; doi:10.1038/s41467-021-23237-2)
Supplement: Supplementary file 1 — Supplementary Information [file 41467_2021_23237_MOESM1_ESM.pdf]

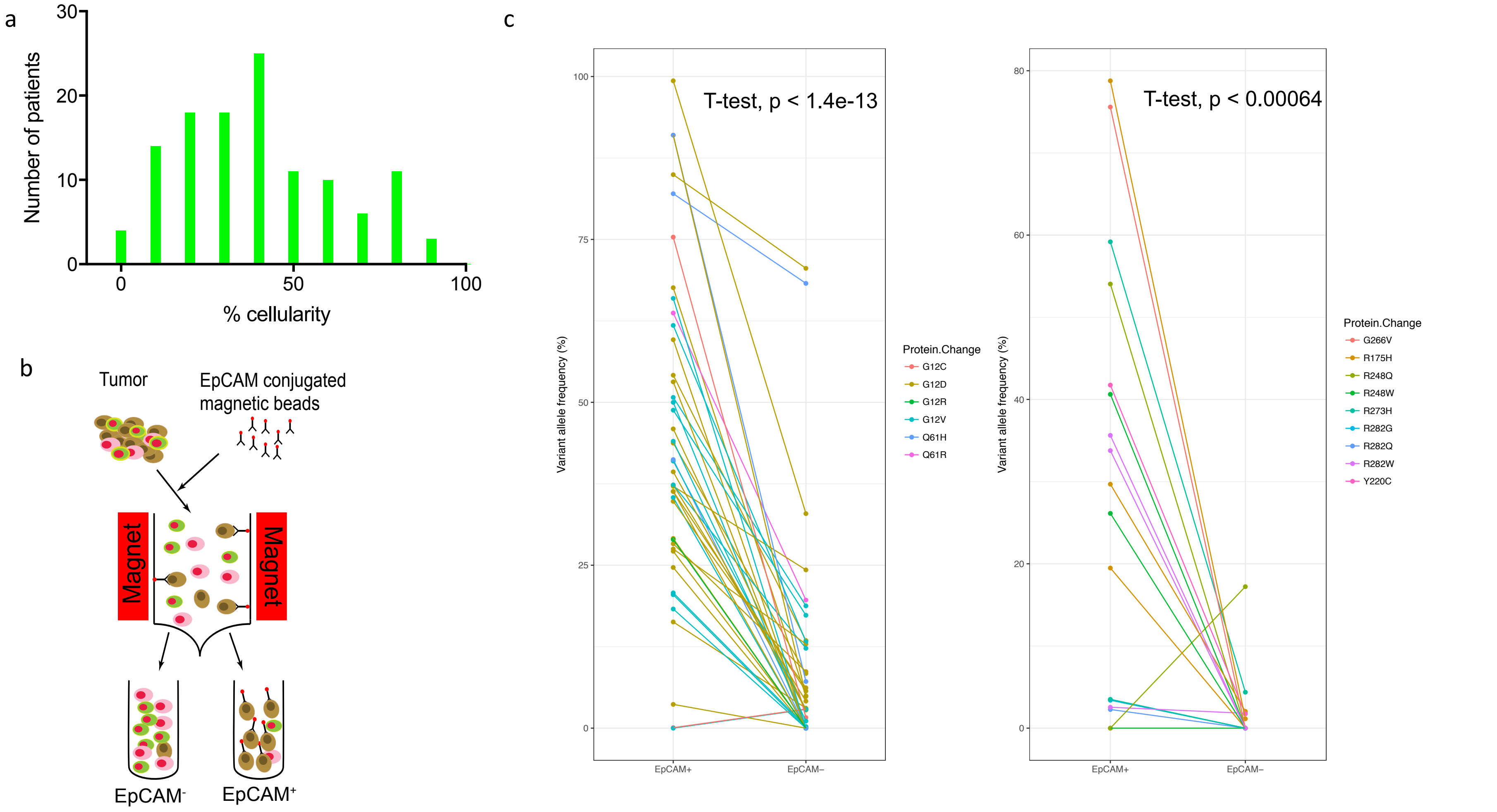

Supplementary Figure 1. Tumor cellularity, isolation of PDAC malignant epithelial cells, and estimation of tumor-cell enrichment by KRAS and TP53 variant allele frequency enrichment analysis **(a)** Tumor epithelial cellularity in the bulk tumors (estimated on frozen sections – at least two sections each of n=120) showing median 40% cellularity with high tumor-to-tumor variability. **(b)** Schematic diagram shows the sorting of PDAC malignant cells from freshly resected tumors using EpCAM antibody-conjugated magnetic beads (drawn by S.C. using Adobe InDesign) **(c)** Canonical variant allele frequencies of *KRAS* (left) and *TP53* (right) comparing the EpCAM$^+$ and EpCAM$^-$ subpopulations from each tumor. The colored lines depict comparative variant allele frequencies in each individual tumor, confirming high level enrichment of mutant alleles in EpCAM$^+$ subpopulations (two tailed unpaired t-test P<0.05) (Source data are provided as a Source Data file).

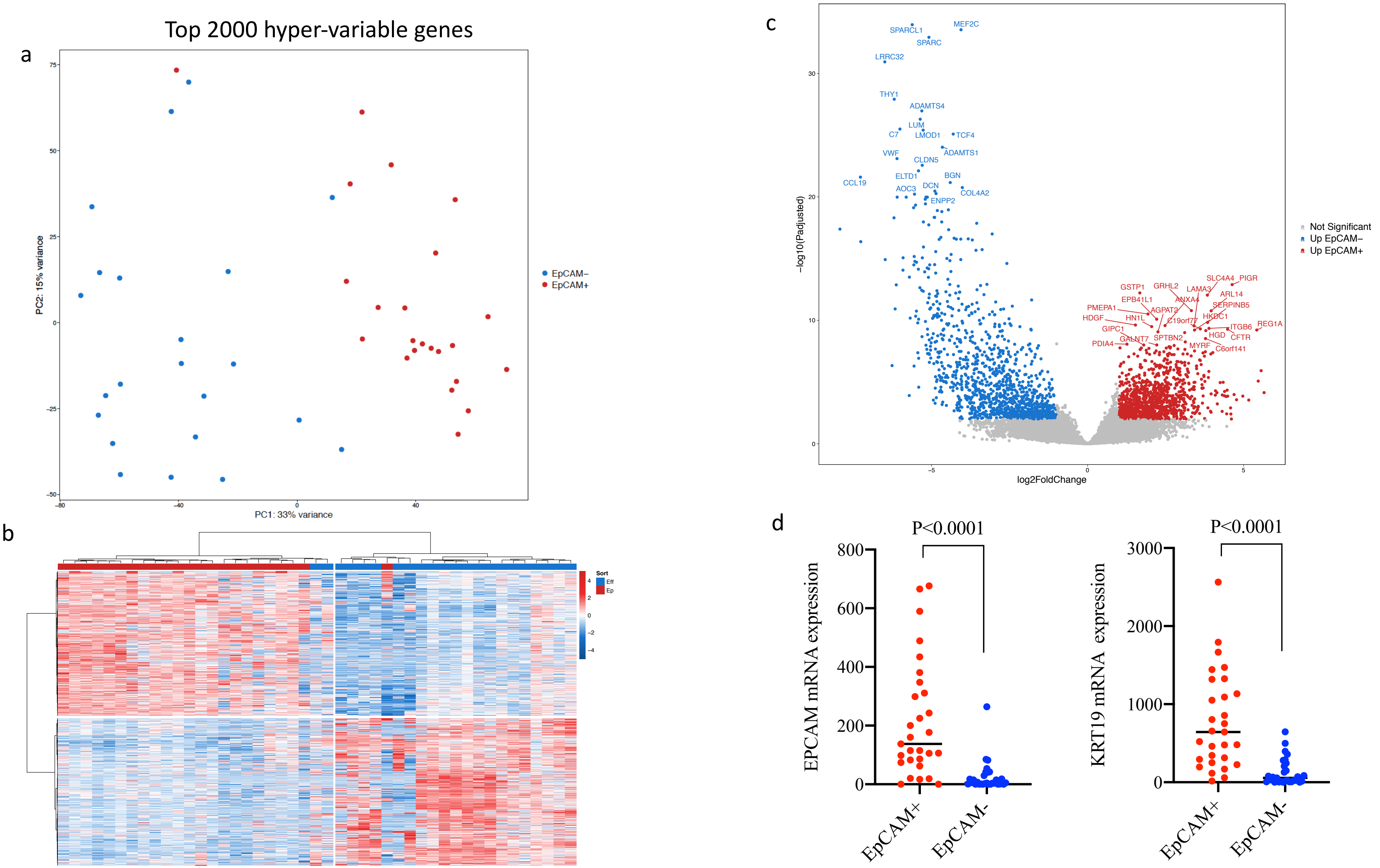

Supplementary Figure 2. Estimation of tumor-cell enrichment by RNA analysis **(a)** Principal Component Analysis of the expression of top 2000 hypervariable genes in EpCAM^{+} and EpCAM^{-} cells from each tumor. **(b)** Heatmap showing differential expression of genes between EpCAM^{+} and EpCAM^{-} cells. **(c)** Volcano plot showing upregulated genes in EpCAM^{+} (red) and EpCAM^{-} (blue) cells. **(d)** Expression of selected epithelial genes *EpCAM* and *KRT19* mRNA in EpCAM^{+} and EpCAM^{-} subpopulations. Statistical tests are unpaired two tailed t-test with P <0.05 is significant, comparing EpCAM^{+} (n=29) and EpCAM^{-} (n=29) subpopulations (Source data are provided as a Source Data file).

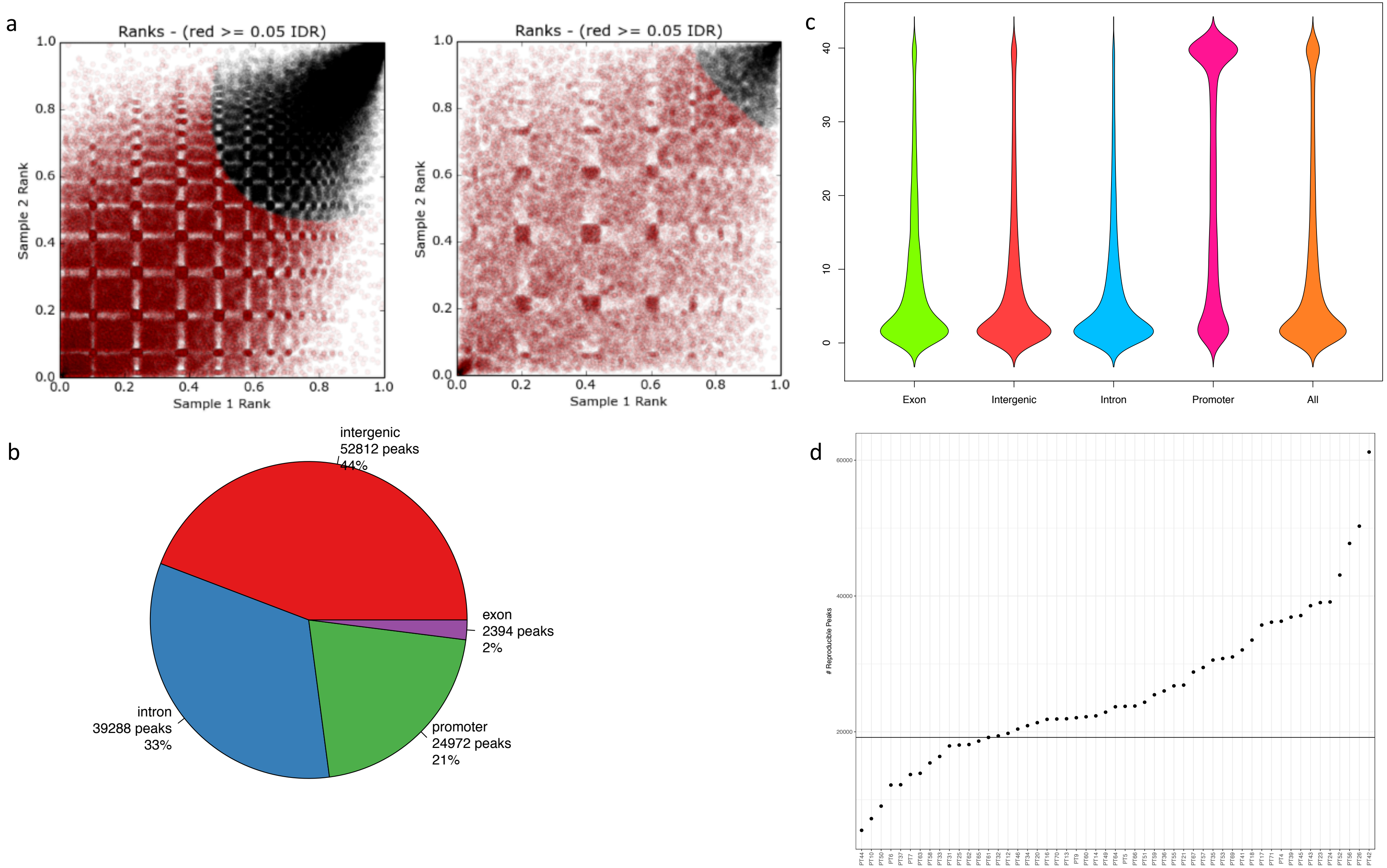

Supplementary Figure 3. Discovery of open chromatin peaks in human PDAC **(a)** Irreproducible Discovery Rate (IDR) depicting representative good quality (left) and a bad quality (right) ATAC-seq libraries. **(b)** Distribution of accessible promoter, intronic, exonic and intergenic peaks, as mapped on gene loci following ATAC-seq. **(c)** Bean plot showing the distribution of the ATAC-seq peaks among patients (n=40). **(d)** Exclusion of the lowest quartile of 14 samples from the complete cohort (n=54) by ranking them on the basis of number of (IDR) reproducible ATAC-seq peaks contributed by each patient, in order to selecting the best quality samples with which to form the global atlas (n=40) (Source data are provided as a Source Data file).

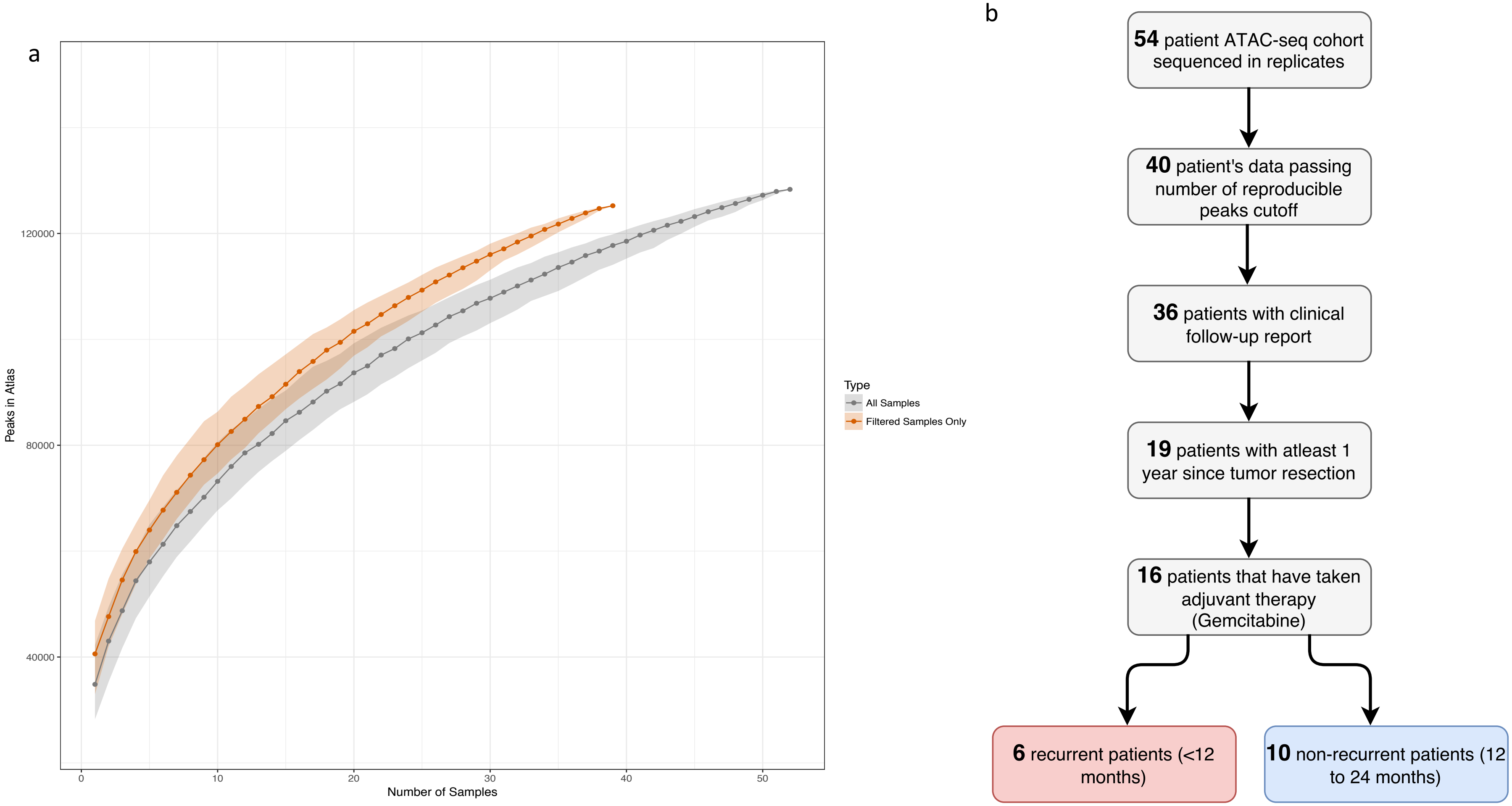

Supplementary Figure 4. Cohort level saturation of the open chromatin peaks in human PDAC **(a)** Cohort-level saturation of the peaks on all the patients (n=54, grey) and the patients included in the global atlas (n=40, orange). **(d)** Flowchart showing selection of patients used for training set (n=16) (Source data are provided as a Source Data file).

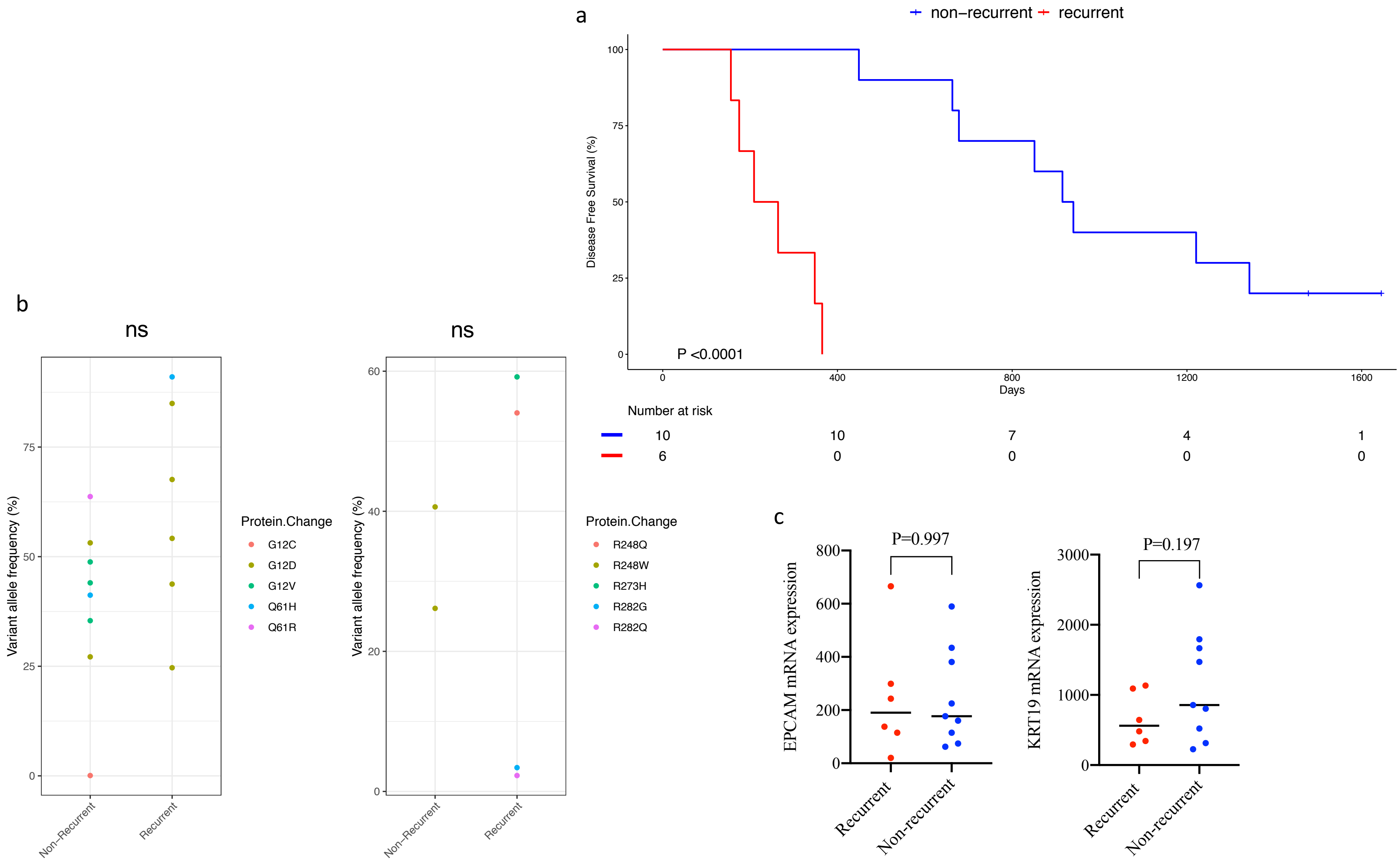

Supplementary Figure 5. Age, sex and malignant epithelial cellularity adjustments of the discovery set of the PDAC cohort (n=16). **(a)** Kaplan-Meier graph showing the segregation of the recurrent (n=6) and non-recurrent (n=10) group of patients with a median 4.15 (min=3.18, and max= 4.75) years of follow up (log rank P<0.0001, HR 0.1579, 95% CI of HR 0.02877 to 0.8665). **(b)** Non-significant differences of KRAS and TP53 variant allele frequencies (different colored dots represent different canonical variant alleles), and **(c)** non-significant differences of EpCAM and KRT19 mRNA expression between the recurrent (n=6) and non-recurrent (n=10) groups (unpaired two tailed t-test with P <0.05 is significant) (Source data are provided as a Source Data file).

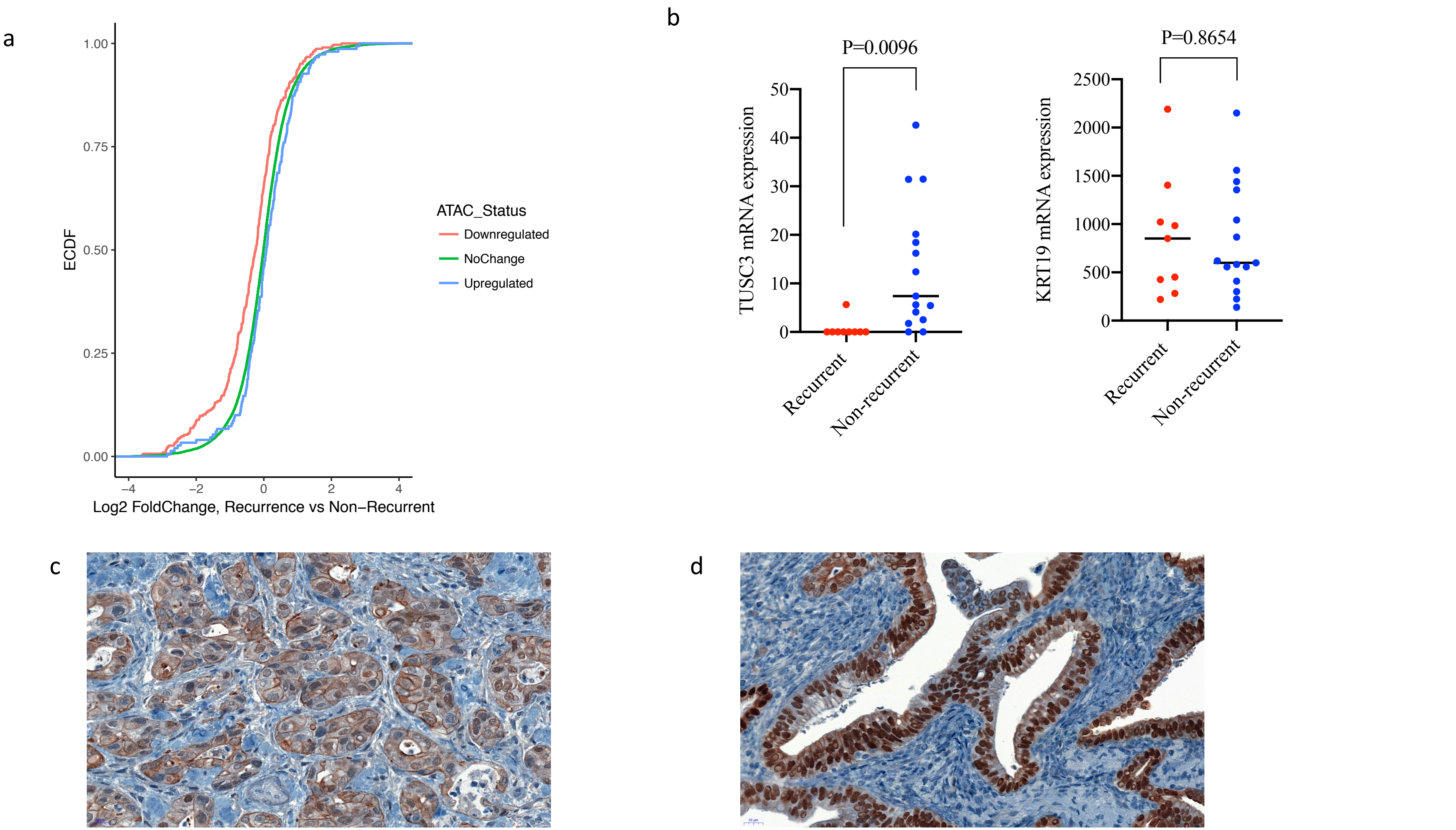

Supplementary Figure 6. Gene expression correlation of differentially represented peaks along with the immunohistochemistry staining of HNF1b. **(a)** Empirical cumulative distribution frequency (ECDF) of expressed genes annotated to ATAC-seq peaks comparing the expression of downregulated (red) and the upregulated (blue) genes with the unaltered (green) set of genes (statistical test is Kolmogorov-Smirnov test). **(b)** Expression of *TUSC3* and *KRT19* mRNA in EpCAM^{+} PDAC malignant cells of recurrent (red) and non-recurrent patients (blue). Statistical tests are unpaired two tailed t-test with $P < 0.05$ is significant, comparing recurrent (n=9) and non-recurrent (n=15) patients. **(c)** Cytoplasmic and **(d)** nuclear staining of HNF1b by immunohistochemistry on the TMA sections. Scale bars are 20µM as displayed at the left bottom corners of all the micrographs (Source data are provided as a Source Data file).

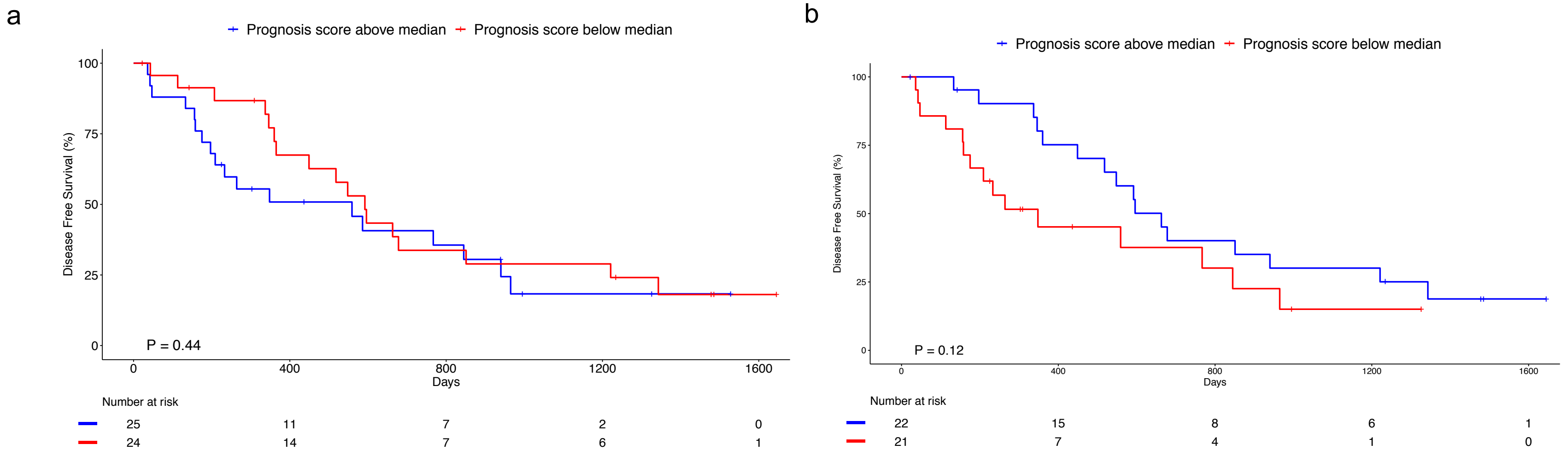

Supplementary Figure 7. Kaplan-Meier graphs showing stratification of the patients by normalized intensities of ATAC-array **(a)** the red peak (RED/(CTRL - CGH)), and **(b)** the difference between blue and red peaks ((BLUE - RED)/(CTRL - CGH)) were not as discriminative as the normalized intensity of the blue peaks (BLUE/(CTRL - CGH) as displayed in Fig 4c. (RED/(CTRL - CGH) log-rank (Mantel-Cox) test P=0.44, HR 0.77, 95% CI 0.3943 to 1.504; and (BLUE - RED)/(CTRL – CGH) log-rank (Mantel-Cox) test P=0.12, HR 1.771, 95% CI: 0.8556−3.664, respectively (Source data are provided as a Source Data file).

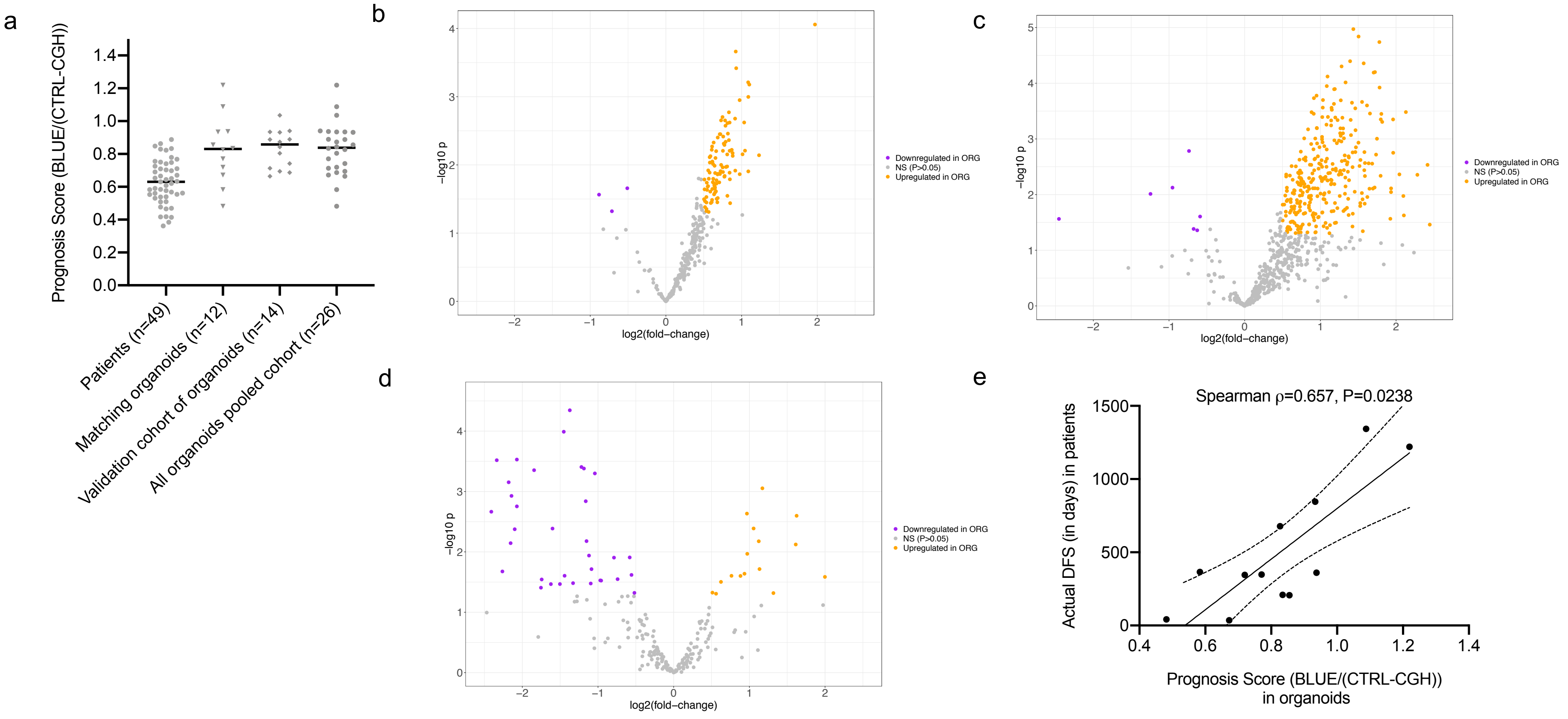

Supplementary Figure 8. Prognosis scores of PDAC patients and patient-tumor derived 3D organoids **(a)** Ranking of the Prognosis Scores derived from ATAC-array on freshly sorted patient tumor cells (n=49), matching patient-derived organoids (n=12), organoids from an independent validation cohort (n=14), and pooled organoid cohort (n=26). **(b)** Volcano plot comparing the Green (CTRL) region intensities between organoids and their tumors of origin showing more significantly open regions in organoids (orange dots on right) than closed regions (purple dots on left) **(c)** Volcano plot comparing the Blue regions between organoids and their tumors of origin showing significantly more open regions in organoids (orange dots on right) than closed regions (purple dots on left) **(d)** Volcano plot comparing the Red region intensities between organoids and their tumors of origin showing significantly more closed regions in organoids (purple dots on left) than open regions (orange dots on right). **(e)** ATAC-array Prognosis Score (BLUE/(CTRL-CGH)) derived from matching organoids correlate with the actual DFS of the patients from which they were derived (Spearman ρ=0.657, 95% CI 0.1150 to 0.8978, P=0.0238, n=12) (Source data are provided as a Source Data file).
