# Supplementary material for: Pancreatic cancer prognosis is predicted by an ATAC-array technology for assessing chromatin accessibility
Source: Nat Commun. 2021 May 24;12:3044. doi: 10.1038/s41467-021-23237-2 (PMC8144607; doi:10.1038/s41467-021-23237-2)
Supplement: Supplementary file 13 — Description of Additional Supplementary Files [file 41467_2021_23237_MOESM13_ESM.pdf]

Title: Supplementary Data 1:
Description: Upregulated and downregulated genes in EpCAM-sorted PDAC malignant cells

Title: Supplementary Data 2:
Description: Patient clinical records (n=54)

Title: Supplementary Data 3:
Description: List of 1092 ATAC-Seq peaks

Title: Supplementary Data 4:
Description: HNF1b TF-binding motif identified to the nearest genes

Title: Supplementary Data 5:
Description: ZKSCAN1 TF-binding motif identified to the nearest genes

Title: Supplementary Data 6:
Description: ATAC-Array cohort (n=49)

Title: Supplementary Data 7:
Description: ATAC-Array HNF1b common cohort (n=39)

Title: Supplementary Data 8:
Description: Organoids that are overlapping with ATAC-Seq/Array patient cohort (n=12)

Title: Supplementary Data 9:
Description: Validation cohort of organoids that are not overlapping with ATAC-Seq/Array patient cohort (n=14)

Title: Supplementary Data 10:
Description: Primers used to prepare ATAC-seq libraries (Buenrostro et al, Nat Method 2013 ref 10 of the manuscript)
