# Supplementary material for: Pancreatic cancer prognosis is predicted by an ATAC-array technology for assessing chromatin accessibility
Source: Nat Commun. 2021 May 24;12:3044. doi: 10.1038/s41467-021-23237-2 (PMC8144607; doi:10.1038/s41467-021-23237-2)
Supplement: Supplementary file 14 — Reporting Summary [file 41467_2021_23237_MOESM14_ESM.pdf]

nature research

Corresponding author(s): Steven D. Leach
Christina S. Leslie

Last updated by author(s): Mar 29, 2021

Reporting Summary

Nature Research wishes to improve the reproducibility of the work that we publish. This form provides structure for consistency and transparency in reporting. For further information on Nature Research policies, see our Editorial Policies and the Editorial Policy Checklist.

Statistics

For all statistical analyses, confirm that the following items are present in the figure legend, table legend, main text, or Methods section.

n/a	Confirmed	
☐	☒	The exact sample size (*n*) for each experimental group/condition, given as a discrete number and unit of measurement
☐	☒	A statement on whether measurements were taken from distinct samples or whether the same sample was measured repeatedly
☐	☒	The statistical test(s) used AND whether they are one- or two-sided<br>*Only common tests should be described solely by name; describe more complex techniques in the Methods section.*
☐	☒	A description of all covariates tested
☐	☒	A description of any assumptions or corrections, such as tests of normality and adjustment for multiple comparisons
☐	☒	A full description of the statistical parameters including central tendency (e.g. means) or other basic estimates (e.g. regression coefficient) AND variation (e.g. standard deviation) or associated estimates of uncertainty (e.g. confidence intervals)
☐	☒	For null hypothesis testing, the test statistic (e.g. *F*, *t*, *r*) with confidence intervals, effect sizes, degrees of freedom and *P* value noted<br>*Give P values as exact values whenever suitable.*
☒	☐	For Bayesian analysis, information on the choice of priors and Markov chain Monte Carlo settings
☒	☐	For hierarchical and complex designs, identification of the appropriate level for tests and full reporting of outcomes
☒	☐	Estimates of effect sizes (e.g. Cohen's *d*, Pearson's *r*), indicating how they were calculated

Our web collection on statistics for biologists contains articles on many of the points above.

Software and code

Policy information about availability of computer code

Data collection	No software was used
Data analysis	ATAC-seq analysis:<br>Raw fastq files were trimmed using trimmomatic (v0.35). The samples were then aligned using bowtie2(v2.2.6). Duplicate read removal was performed using MarkDuplicates (v2.9.0). Peak calling was performed using MACS2 (v2.1.0). IDR (irreproducible discovery rate) was used to identify reproducible peaks from the duplicate libraries for each sample. Read counting was performed using GenomicRanges package in R. FIMO (v5.0.0) and glmnet (v2.0-16) were used for differential motif analysis.<br><br>ATAC-array analysis:<br>ATAC-array data analysis was performed following the standard array-CGH analysis pipeline of Agilent Technologies Inc. Agilent's commercially available Feature Extraction (FE) software (FE Version 12.1.0.3) extracted the output text files containing the probe intensities. The median normalized intensities of all the probes for all the ATAC differential regions, control regions and CGH backbone regions were outputted along with the gene annotations for downstream analyses.

For manuscripts utilizing custom algorithms or software that are central to the research but not yet described in published literature, software must be made available to editors and reviewers. We strongly encourage code deposition in a community repository (e.g. GitHub). See the Nature Research guidelines for submitting code & software for further information.

Data

Policy information about availability of data

All manuscripts must include a data availability statement. This statement should provide the following information, where applicable:

- Accession codes, unique identifiers, or web links for publicly available datasets
- A list of figures that have associated raw data
- A description of any restrictions on data availability

Raw data have been deposited in GEO dbGaP under controlled access and the links are provided in the manuscript. DATA ACCESS: Processed ATAC-seq and RNA Quant-seq data has been deposited to GEO (GSE124229 and GSE124230, respectively). Raw data has been made accessible through controlled-access dbGaP portal (phs002394.v1.p1). The ATAC-array raw data are uploaded in the following GitHub link along with the analysis codes

CODE AVAILABILITY: All codes are available through the following GitHub link
https://github.com/hchintalapudi/ATAC-array

Field-specific reporting

Please select the one below that is the best fit for your research. If you are not sure, read the appropriate sections before making your selection.

[x] Life sciences [ ] Behavioural & social sciences [ ] Ecological, evolutionary & environmental sciences

For a reference copy of the document with all sections, see nature.com/documents/nr-reporting-summary-flat.pdf

Life sciences study design

All studies must disclose on these points even when the disclosure is negative.

Sample size	54 primary PDAC samples were collected for ATAC-sequencing in a progressive cohort (from 2015 to 2017) as available to us. We performed saturation analysis to estimate incremental new peak discovery associated with stepwise increases in sample size and confirmed that a sample size of n=54 and n=40 both approached saturating coverage (as described in methods and presented in Supplementary Figure 4a).<br><br>Another independent cohort of tissue microarray for 97 PDAC tumors (with three replicate cores from each of the tumors) containing 45 short-term and 52 long-term survivor patients was used to validate HNF1b and ZKSCAN1 immunostainings. This cohort was originally developed in the Leach lab and published (Balachandran et, al, Nature 2017)<br><br>Another independent cohort of 26 organoids were used to validate the ATAC-array results. 12 of them were matching with the tumors of origin where ATAC-seq was performed, and the rest 14 organoids were generated from completely independent PDAC tumors (resected). The sample size of organoids were selected based on availability in our repository.<br><br>The inclusion and exclusion criteria are as follows: inclusion: Histologically confirmed Pancreatic Ductal Adenocarcinoma, treatment-naive and surgically resected followed by Gemcitabine/Abraxane adjuvant chemotherapy treatment regimen. Exclusion: Neo-adjuvant chemotherapy treated patients who have undergone surgical resection. Organoids cohort also followed the exactly the same inclusion and exclusion criteria.
Data exclusions	IDR (irreproducible discovery rate), as described in the methods section and presented in Supplementary Figure S3a and S3d, was used to identify reproducible peaks from the duplicate libraries for each sample (IDR < 1 x 10−2). 14 patients from the bottom quartile of reproducible peaks were excluded to select the IDR-qualified (best quality samples) contributed reproducible peaks.
Replication	Single-cell suspension from every patient (n=54) was divided into two aliquots and transposition reactions were performed independently on the two aliquots followed by independent library preparation and ATAC-sequencing to ensure reproducibility.
Randomization	Patients were allocated into recurrent or non-recurrent based on whether they recurred within 1 year, for the discovery of 1092-ATAC-seq differential signature. ATAC-array was done randomly on each patient (n=49) followed by Kaplan-Meier analysis on a 4.15 year of median follow-up records
Blinding	Blinding was not relevant for ATAC-seq analysis since the samples were collected in a progressive cohort and the recurrence status was unknown at the time of sample collection. However, immunohistochemical (IHC) and immunofluorescence (IF) analyses were blinded.

Reporting for specific materials, systems and methods

We require information from authors about some types of materials, experimental systems and methods used in many studies. Here, indicate whether each material, system or method listed is relevant to your study. If you are not sure if a list item applies to your research, read the appropriate section before selecting a response.

Materials & experimental systems

n/a	Involved in the study	
	☒	Antibodies
☒		Eukaryotic cell lines
☒		Palaeontology and archaeology
☒		Animals and other organisms
	☒	Human research participants
☒		Clinical data
☒		Dual use research of concern

Methods

n/a	Involved in the study	
☒		ChIP-seq
☒		Flow cytometry
☒		MRI-based neuroimaging

Antibodies

Antibodies used

EpCAM-conjugated magnetic beads (Milteney Biotech Cat# 130-061-101), anti-ZKSCAN1 (Sigma, cat#HPA006672, 0.5ug/ml), biotinylated goat anti-rabbit IgG (Vector labs, cat#PK6101), anti-HNF1b (Sigma, cat#HPA002085, 1ug/ml), anti-CK19 (Abcam, cat#ab52625, 0.02ug/ml), Tyramide Alexa Fluor 488 (Invitrogen, cat# B40953), and Tyramide Alexa 568 (Invitrogen, cat# T20948)

Validation

All antibodies as mentioned above are commercially available and they are validated by the manufacturer

Human research participants

Policy information about studies involving human research participants

Population characteristics

Treatment-naive surgically resected pancreatic ductal adenocarcinoma (PDAC) patients. No confounding effects of Age, sex and epithelial cellularity were identified

Recruitment

All samples were randomly collected to avoid any patient selection bias, starting from Sept 2015 to March 2017 and followed up until Nov 2017 for the discovery analysis, and until July 2020 for the full cohort. The median age of the PDAC patients was 68 years, ranging from 50 to 89 years, male 25, and female 29, as listed in the Supplementary Table S2. All tumor samples were treatment-naïve surgically resected primary pancreatic ductal adenocarcinomas. Patients treated with neoadjuvant therapy were excluded. Only histologically-confirmed PDAC tumors were included in the study. .

Ethics oversight

All tissues were collected at Memorial Sloan Kettering Cancer Center (MSKCC) following a study protocol approved by the MSKCC Institutional Review Board, and also by the Dartmouth Institutional Review Board. Informed consent was obtained from all patients.

Note that full information on the approval of the study protocol must also be provided in the manuscript.
